# Improving the external validity of clinical trials: the case of multiple chronic conditions

**DOI:** 10.15256/joc.2013.3.27

**Published:** 2013-12-24

**Authors:** Fortin Martin, Smith M. Susan

**Affiliations:** ^1^Department of Family Medicine, Université de Sherbrooke, Sherbrooke, Quebec, Canada; Centre de santé et de services sociaux de Chicoutimi, Chicoutimi, Quebec, Canada; ^2^HRB Centre for Primary Care Research, Department of General Practice, Royal College of Surgeons, Dublin, Ireland

**Keywords:** external validity, internal validity, clinical trials, comorbidity, multimorbidity, multiple chronic conditions

## Abstract

The U.S. Department of Health and Human Services vision and strategic framework on multiple chronic conditions (MCCs) incorporates recommendations designed to facilitate research that will improve our knowledge about interventions and systems that will benefit individuals with MCCs (or multimorbidity). The evidence base supporting the management of patients with MCCs will be built through intervention trials specifically designed to address multimorbidity and identification of MCCs in participants across the clinical trial range. This article specifically focuses on issues relating to external validity with specific reference to trials involving patients with MCCs. The exclusion of such patients from clinical trials has been well documented. Randomized control trials (RCTs) are considered the “gold standard” of evidence, but may have drawbacks in relation to external validity, particularly in relation to multimorbidity. It may, therefore, be necessary to consider a broader range of research methods that can provide converging evidence on intervention effects to address MCCs. Approaches can also be taken to increase the usefulness of RCTs in general for providing evidence to inform multimorbidity management. Additional improvements to RCTs would include better reporting of inclusion and exclusion criteria and participant characteristics in relation to MCCs. New trials should be considered in terms of how they will add to the existing evidence base and should inform how interventions may work in different settings and patient groups. Research on treatments and interventions for patients with MCCs is badly needed. It is important that this research includes patient-centered measures and that generalizability issues be explicitly addressed.

Journal of Comorbidity 2013;3:30–35

## Introduction

Enhancing the external validity of clinical trials is one of the priority areas identified in the initiative launched by the U.S. Department of Health and Human Services (HHS) to strengthen efforts to address the challenge of the rising prevalence of multiple chronic conditions (MCCs), or multimorbidity, in the population [1]. This article specifically focuses on issues relating to external validity of evidence, with specific reference to trials involving patients with MCCs. We also consider some potential solutions to address the problem of low external validity of clinical trials for these patients. There are two distinct scenarios in relation to trials and multimorbidity. The first occurs where a trial has been specifically designed to test an intervention that addresses MCCs [2, 3] and the second relates to the likelihood that participants in most clinical trials are likely to have multimorbidity to some extent and the question is how generalizable the results of that trial are to all patients with MCCs. The challenge inherent to research in multimorbidity is that there is heterogeneity within the group. At its simplest level, MCCs includes patients with clear comorbid conditions, but this then expands out to more general multimorbidity, in which there is no prespecified index condition and there are multiple possible combinations and permutations of conditions and condition severity [4].

External validity, sometimes also referred to as generalizability, describes the applicability of interventions in settings beyond the original study. However, determining the external validity of a study is more difficult than determining its counterpart, internal validity. Internal validity describes the degree to which the design and conduct of a trial reduces the possibility of bias within that trial, and it can be judged using the list of items in guidelines on trial methodology and reporting, such as the CONSORT (Consolidated Standards of Reporting Trials) statement [5]. Determining whether the results of a trial are valid for patients other than those in the original study population requires detailed information on participant characteristics, clinical expertise, and an understanding of healthcare systems and settings, rather than statistical or methodological expertise [6]. External validity is considered a complex concept [6, 7], and has been defined as “the overarching descriptor for all aspects of the design and performance that impact on the external usefulness of the result of a trial, independent of the ‘internal validity’ of the trial” [6].

General aspects that may impact on the external validity of trials can be divided into system and patient factors. Patient factors are reflected in the selection of patients and the potential differences between enrolled patients and the general patient population [6]. System factors include the setting of the trial, differences between trial protocol and routine practice, and potential impact of the intervention on service organization and costs for patients and providers, which, in turn, depend on financing within health systems. While there is a clear need for further studies of interventions in patients with multimorbidity [8], this paper specifically focuses on issues relating to external validity which are particularly relevant for those with MCCs.

## Challenges to external validity

One of the main challenges to external validity of trials is that most clinical trials targeting chronic conditions would find a high number of patients with MCCs during the recruitment process [9]. This situation applies particularly to trials involving patients in primary care settings because it is known that patients with MCCs constitute the majority of patients in this setting [10]. Nevertheless, the potential exclusion of such patients from clinical trials has been well documented [11, 12]. Clinical trials should provide evidence to inform policy and practice regarding new treatments and interventions for all patients. This can be addressed either by specifically designing trials to test interventions that address multimorbidity or, in these trials, reporting of participant characteristics and intervention settings and components allowing for an interpretation of external validity. It is more difficult, in general, in clinical trials, where exclusion of patients with MCCs means that important information about the proper use of a treatment or intervention for those patients is not available. In other words, the exclusion of patients with MCCs limits the external validity of clinical trials for these patients [13–16]. However, many of these trials may have included patients with MCCs, but not identified them as such, and this is a problem frequently observed in reports of clinical trials where there is low reporting of data around participant characteristics that could have informed external validity [13, 17]. For example, in a randomized trial of peer support for patients with type 2 diabetes, a secondary analysis of characteristics of all included patients indicated that 90% had MCCs, with 25% having four or more conditions. Within the cohort of 424 patients, there were 189 unique conditions [18]. This highlights the importance of fully reporting participant characteristics to facilitate a complete and correct interpretation of the generalizability of the trial’s results. Table 1 highlights other potential challenges to external validity, including whether patients with MCCs will agree to participate in clinical trials as they may already be overburdened by treatment and healthcare attendances [19]. Additionally, interventions may have a single-condition focus, making them of less interest and use to patients with MCCs, or there may be costs involved to intervention participation that act as a deterrent to those already experiencing high health service utilization and costs [20, 21].

When seeking to find ways to improve the external validity of clinical trials for patients with MCCs, one soon encounters a barrier relating to the potential imbalance between internal and external validity in randomized controlled trials (RCTs) [22]. It can be argued that internal validity is a prerequisite for external validity as there is no point in trying to generalize evidence from a poor-quality trial [23]. RCTs are considered the “gold standard” of evidence because of their ability to reduce selection bias and other factors that could bias outcomes, so researchers usually aim to enhance internal validity by having replicable and clear patient inclusion criteria that may lead to the exclusion of medically complex subjects. However, this will likely be at the cost of reducing external validity, which may initially seem to be of secondary importance. Results regarding the effectiveness of the intervention tested in an RCT with high internal validity will be more robust, but they will probably provide limited evidence to support care for patients with MCCs found in real life. A consequence of poor external validity of clinical trials is that clinical practice guidelines, which are often based primarily on expert reviewof the hierarchy of evidence from relevant clinical trials, can have limited utility for patients with MCCs or may lead to inappropriate use of interventions in these patients [24, 25].

## Potential solutions

In the initiative launched by the HHS, one of the objectives identified within the goal of facilitating research to fill knowledge gaps was to enhance the external validity of clinical trials. To meet this goal, four objectives were outlined: (i) to develop methods to assess the inclusion of individuals with MCCs in clinical trials; (ii) to improve the external validity of trials by ensuring that individuals with MCCs are not unnecessarily excluded; (iii) to ensure that individuals with MCCs are not unnecessarily excluded from clinical trials for the approval of prospective drugs and devices; and (iv) to assess and strengthen postmarketing surveillance for potential intervention-related adverse effects and poor outcomes among individuals with MCCs. To increase the external validity of trials for individuals with MCCs is a challenging task and can be considered in individual areas.

### Consider alternative and complementary designs for trials in MCCs

Some possible solutions have been proposed to enhance the external validity of RCTs in general [22, 26–28]. Within this ongoing discussion, Kaplan and colleagues [27] made an interesting point when they said, “evidence based medicine has made a leap from considering RCTs to be a high standard to being the only standard”. This suggests that we may need to reconsider our over-reliance on RCTs to measure intervention effects and make a greater use of alternative forms of evidence, particularly for patients with MCCs. Use of mixed research methods, i.e. combining experimental designs with parallel qualitative process evaluations, can provide converging evidence on intervention effects and may be a good way to address the problem of external validity. The accumulated evidence about an intervention would be gathered from a combination of RCTs and other methods that would provide more information to support the judgements related to external validity for patients with MCCs. Alternative designs could also be considered [3]. For example, this could include crossover trials with patients serving as their own controls with periods of intervention and no intervention distributed at random where interventions are not thought to have sustained effects beyond their delivery. Nevertheless, alternative study designs are not the panacea as they may be compromised by bias and all new trials should be considered in terms of how they will add to the existing evidence base. This emphasizes the importance of conducting trials using designs that will ultimately be incorporated in new updates of systematic reviews [29].

### Consider external validity when designing and reporting current RCTs

In pragmatic, randomized clinical trials, the aim is to establish the effectiveness rather than the efficacy of interventions. Participants who are similar to patients typically seen by clinicians in routine practice are therefore included to achieve a balance between pragmatism and individualization of interventions and generalizability. This addresses the HSS strategy on the inclusion of individuals with MCCs in clinical trials. Funders of RCTs could emphasize the need to maximize external validity of RCTs in relation to MCCs as an important condition for financing studies, as suggested in Strategy 2 of the HHS. RCTs would have greater external validity if researchers tried to find an adequate balance between the demonstrated benefit in ideal conditions in samples of patients with no coexisting health problems, and benefit in the broader population under less ideal circumstances [27]. This balance should also be reflected in clinical trials for the approval of prospective drugs and devices, as outlined in Strategy 3 of the HHS.

### Consider alternative sources of evidence

Postmarketing surveillance of drugs as well as collection and analysis of administrative clinical data about interventions being applied to patients with MCCs would also provide valuable evidence. This is in agreement with the HHS strategy on strengthening post-marketing surveillance for potential intervention-related adverse effects and poor outcomes among individuals with MCCs. In some situations, expert opinions about the management of patients with MCCs passed down through consensus guidelines may be the only available source of “evidence”. Qualitative methods, treatment fidelity analysis, and epidemiological data, including prospective cohort studies, are other examples of additional sources of information, particularly around potential harm, as this generally requires larger sample sizes than generally found in RCTs. We believe that the acceptance of a new drug in the market as well as a new intervention in practice should rely on multiple sources of evidence incorporating data on patients with MCCs and not just be reliant on evidence from RCTs. This would help to ensure that individuals with MCCs are not unnecessarily excluded from evidence base for the approval of drugs and devices, as outlined in Strategy 3 of the HHS.

### Consider secondary data analysis of existing trial evidence

Another approach to enhance the current evidence base to support management of MCCs would be to conduct secondary analyses of existing trial databases using available data relating to MCCs. This could be conducted by increasing trial datasets that are made available for public access, particularly for trials funded through government agencies [30]. This would provide further evidence of the potential effectiveness of the intervention in patients with MCCs. These analyses could be based on existing information on chronic conditions, but if this is not available, there may be other information in trial databases, such as counts of prescribed medicines, which can be a good proxy for MCCs [31].

The issues related to the external validity of clinical trials and possible solutions are summarized in Table 1. The key issue is that recruitment of patients with MCCs to trials will make RCTs more informative about the health impacts of a new intervention for the broader population.

## Concluding remarks

We have addressed the need to consider patients with MCCs, both in the evaluation of trials testing new health interventions generally and in trials testing interventions specifically designed to address multimorbidity. So far, the majority of research on interventions in MCCs has focused on organizational aspects of health care [32]. Further research on treatments for patients with MCCs is badly needed [2, 32]. However, to do specific research on MCCs, we have to think about different ways of developing and adapting the evidence base to enhance clinical care and patient outcomes. We should also focus the interventions on patient-identified problems, which will mean involving patients and care providers in decision-making about which interventions to test [26]. Clinicians need evidence-based guidelines to support treatments, and these need to be informed by trials of treatments and interventions that reflect the priorities of patients and healthcare providers and can be generalized to support care for those living with and managing MCCs.

## Figures and Tables

**Table 1:** Issues related to the external validity of trials involving patients with multiple chronic conditions (MCCs).

Issue	Potential solution
Patient factors	
Lack of clarity around participant inclusion and definitions	Need to have clear definitions of MCCs – whether derived from health record or patient report; condition counts vs. condition severity scores and whether restricted lists of included conditions applied
Participants with MCCs are less likely to consent to participate if disease burden is too high or if in poor health	Consider minimizing burden of participation in intervention
Participants with MCCs may see single-condition trial as being less relevant to them	Consider interventions that are not condition specific or that address specific concerns of MCCs, such as functional or physical performance
Prespecified ancillary analyses taking into account patients’ heterogeneity	Consider preplanning subgroup analyses based on condition counts, severity, and condition combinations
Age of participants with MCCs	Interventions for middle-aged adults with MCCs who are still working may need to be quite different from those for older participants
System factors	
Chronic disease interventions designed around single conditions	Consider more generalized interventions, such as medicines management or support for self-management
Setting: primary vs. specialty care setting	Patient populations and clinicians will be quite different in both settings, but this may be less of an issue for patients with MCCs who commonly attend multiple healthcare providers
System financing issues	Avoid interventions that increase direct or indirect costs to patients or providers
Organizational setting	Intervention embedded in the system that reflects usual care for patients
